# Persistent Winking Coronary Sign in a Case of Post-myocardial Infarction Ventricular Septal Rupture After Device Closure

**DOI:** 10.7759/cureus.39167

**Published:** 2023-05-17

**Authors:** Souvik Sarkar, Gajendra Agrawal, Satish Khadse, Anuj Chaturvedi

**Affiliations:** 1 Respiratory Medicine, Datta Meghe Institute of Higher Education and Research, Wardha, IND; 2 Cardiology, Datta Meghe Institute of Higher Education and Research, Wardha, IND

**Keywords:** percutaneous arterial closure device, ventricular septal rupture, coronary angiography, post-myocardial infarction ventricular septal rupture, winking coronary sign

## Abstract

A winking coronary sign refers to the partial collapse of an artery situated over the ventricular septal rupture during systole and refilling of the same during diastole, which is seen as phasic filling and disappearance of the arterial segment during coronary angiography. In this article, we discuss the case of a patient who reported to the emergency department of a tertiary care hospital in central India with myocardial infarction of the anterior wall. Two-dimensional echocardiography and coronary angiography revealed ventricular septal rupture. The patient was promptly managed by a percutaneous coronary angiography and interventricular septal device closure. Even after the defect closure, the winking coronary sign persisted on coronary angiography, and the patient was then discharged in stable condition.

## Introduction

One of the rarest and most fatal complications of myocardial infarction (MI) is ventricular septal rupture (VSR). A winking coronary sign refers to the partial collapse of an artery situated over the VSR during systole and refilling of the same during diastole, which is seen as phasic filling and disappearance of the arterial segment during coronary angiography. For this structural abnormality, percutaneous defect closure may represent an alternative to surgical closure, which is already considered to be the gold standard. Percutaneous device closure has the advantage of immediately reducing the shunt, which prevents further hemodynamic instability. Here, we present a clinical case of MI where two-dimensional (2D) echocardiography revealed a VSR and coronary angiography showed a winking coronary sign, which was treated with a device closure for the VSR. Even after the correction of the septal defect, there was a persistent winking coronary sign seen in the check angiography of the patient.

## Case presentation

A 65-year-old female presented to the emergency medicine department with complaints of chest pain and breathlessness for two months, which had worsened over the last six hours. She was a known hypertensive for the past four months. On examination, the axillary temperature was 98.6°F, pulse was 106 beats/minute, blood pressure was 104/74 mmHg, and SpO_2_ was 96% on room air. On further examination, the extremities were found to be cold, and there was an increased jugular venous pressure. On auscultation, a pan-systolic murmur was heard in the left parasternal region, and other systems were found to be normal.

**Figure 1:**
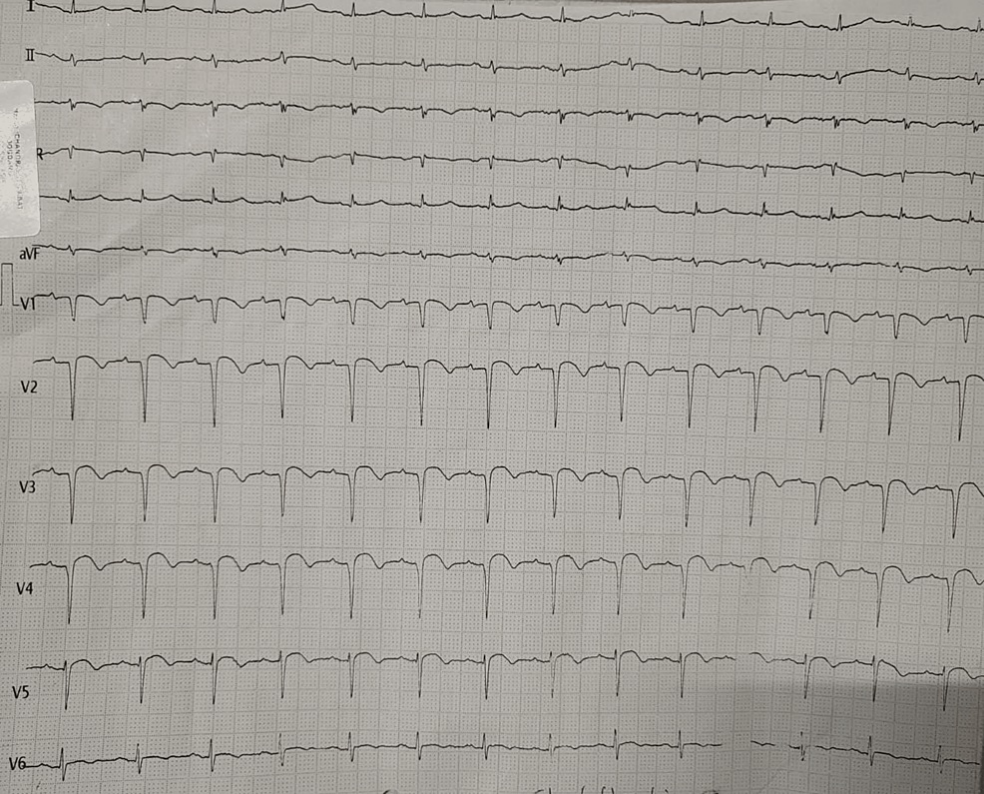
Electrocardiogram showing ST-segment elevation from V1 to V5 and T-wave inversion in leads V1 to V6.

An electrocardiography was done which showed ST elevation from V1 to V5 and T-wave inversion in leads V1 to V6 (Figure 1).

**Figure 2:**
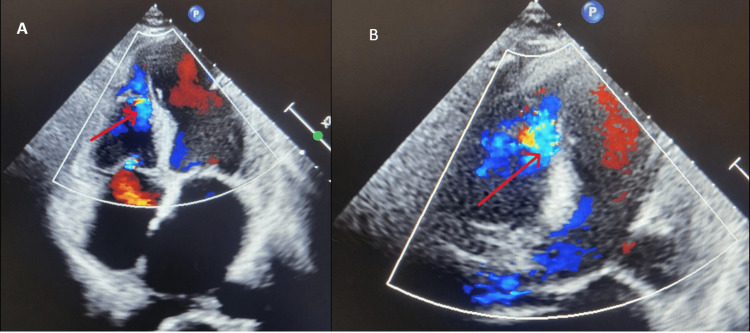
(A) Apical four-chamber view of the echocardiographic image showing a jet across the distal interventricular septum (ventricular septal rupture). (B) Akinetic distal interventricular septum and lateral wall with the ventricular septal rupture.

A bedside 2D echocardiography was performed which revealed an apical VSR of 8-10 mm with severe left ventricular systolic dysfunction. In addition, mild tricuspid regurgitation was noted. The distal interventricular septum and apical lateral wall were akinetic, and the left ventricular ejection fraction was approximately 30% (Figure 2).

**Figure 3:**
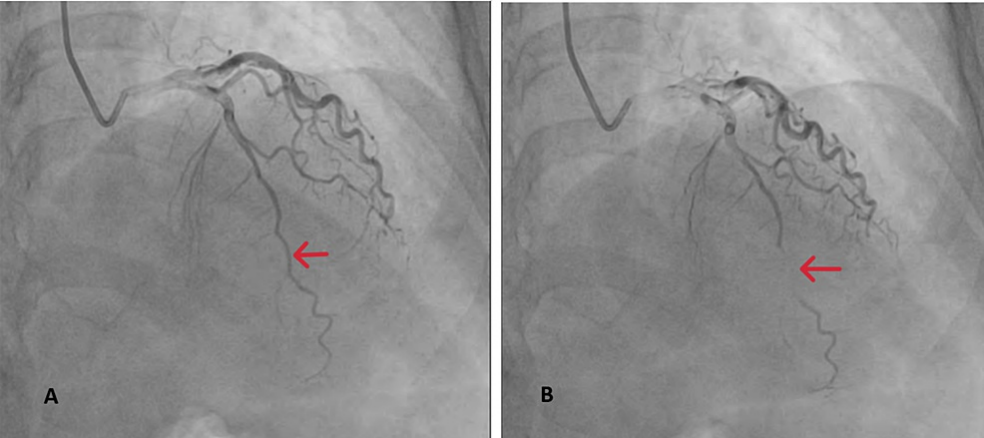
(A) Coronary angiography in the anteroposterior cranial view of the patient showing filling of the left anterior descending artery during diastole. (B) Obliteration of the left anterior descending vein during systole in the anteroposterior cranial view of coronary angiography (winking coronary sign).

The patient was immediately shifted to the cath lab for coronary angiography, which showed a narrowed left anterior descending artery (LAD) with VSR. A winking coronary sign was observed in the distal portion of the LAD, with all other vessels remaining normal (Figure 3).

**Figure 4:**
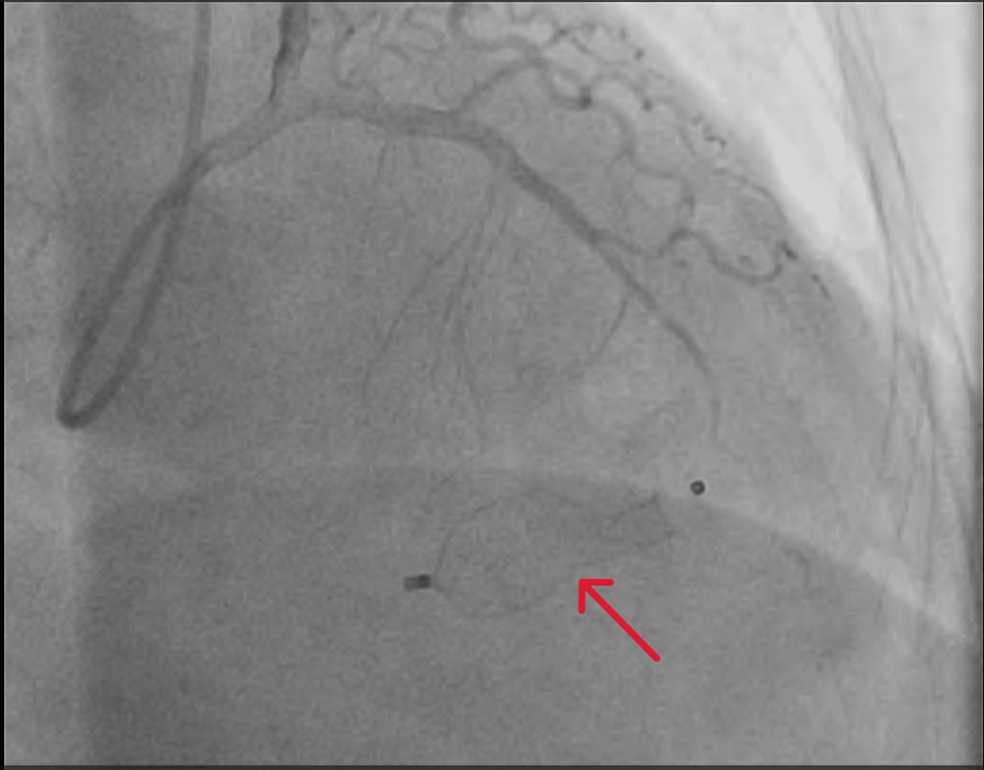
Angiographic image showing the trans-septal placement of the device after closure of the ventricular septal rupture.

The LAD was recanalized and a device closure for the VSR was done uneventfully, after which the blood flow across the VSR was disrupted completely (Figure 4).

## Discussion

**Figure 5:**
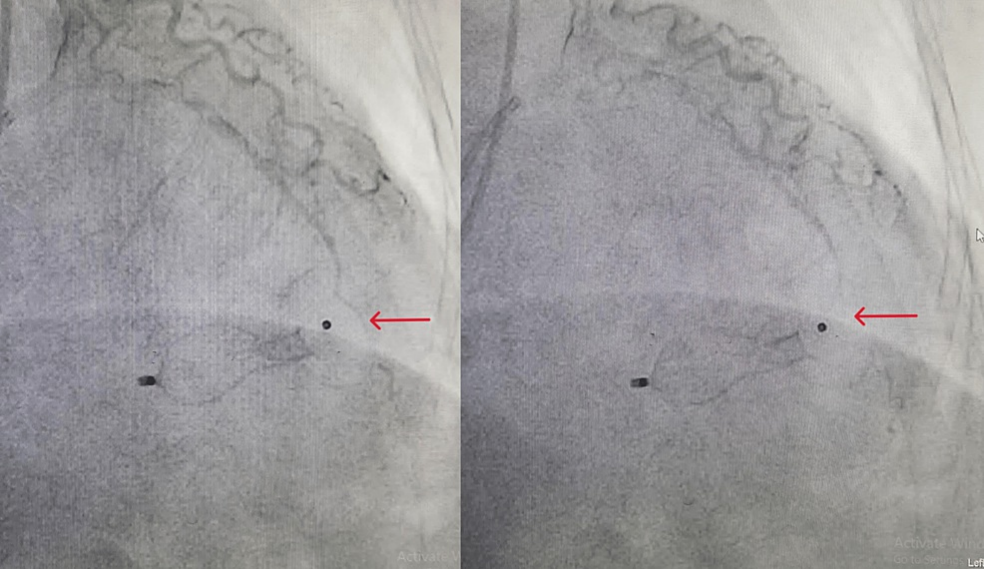
Persistent winking coronary sign seen in the right anterior oblique cranial angiographic view after device closure of ventricular septal rupture.

A winking coronary sign is characterized as a temporary partial blockage of the artery overlaying the VSR during ventricular systole and a near-normal filling during diastole. Early detection of this sign on coronary angiography can aid in the quick diagnosis of VSR and rapid appropriate care can be taken for the same, both of which improve prognosis [1]. The winking coronary sign suggests that there is a loss of myocardium beneath the coronary artery, i.e., LAD in this case, during systole, part of the coronary artery dips down and is squeezed by the underlying damaged myocardium, leading to loss of blood flow in that portion of the coronary artery. A similar situation occurs in myocardial bridging as well where the epicardial artery takes an intramuscular course. According to a study conducted by Sharma et al., winking coronary sign has a sensitivity and specificity of 67.86% and 100%, respectively, for diagnosing a post-MI VSR [1]. The American College of Cardiology and the American Heart Association state that emergency surgery is required regardless of a patient’s hemodynamic status [2]. However, those with stable hemodynamics, favorable anatomy, and no signs of organ failure tend to have better outcomes. However, the ideal time for definitive surgery is still unknown. The European Society of Cardiology recommends delayed surgical treatment in patients who respond initially to aggressive conservative management, such as in our case [3]. By giving the affected tissue time to organize and strengthen, percutaneous closure may provide crucial time that helps an effective surgical repair. A treatment strategy for VSR was proposed by Jones et al. [4] based on hemodynamics and defect architecture. A thorough analysis of the anatomy of the defect is crucial to reduce the chance of procedural failure. In cases where the risk of surgery is too great for the patient, percutaneous closure may be an option. According to the GUSTO-I (Global Utilization of Streptokinase and Tissue Plasminogen Activator for Occluded Coronary Arteries) trial, post-MI VSR patients who were managed conservatively (without a surgical closure of the defect) had high mortality of more than 90% in 30 days. VSR continues to be a highly dangerous MI complication despite significant advancements in device technology and implantation [5]. The preferred course of treatment for patients with post-MI VSR still remains a delayed surgery. Therefore, a closure strategy should be developed as soon as post-MI VSR is identified. In the event that hemodynamic stability is achieved, delaying surgery (i.e., more than 21 days following the identification of post-MI VSR) can have a mortality rate of 20% as opposed to 60% during the first 24 hours [6]. Despite the fact that surgical correction is the gold standard to rectify the defect, percutaneous closure may be a useful therapeutic alternative, which has the benefit of fast shunt reduction and can restrict hemodynamic deterioration [7]. Our team decided to undertake percutaneous closure of the septal rupture due to the patient’s fragility and the immediate surgery’s unacceptable mortality risk. In our patient, although a device closure was done for the VSR, a persistent winking coronary sign was seen in the post-procedure angiographic images (Figure 5).

## Conclusions

A patient with acute MI should always be screened for a VSR, which is a deadly complication. Timely recognition and a percutaneous device closure can help in improving the fatality caused due to post-MI VSR. In our case, although the defect had been closed by a device and the VSR flow was interrupted after the treatment, a persistent winking coronary sign was noticed on angiography. Even though there is the persistence of the winking coronary sign, we do not anticipate any problems in the future. It is always advisable for such patients to be in regular follow-ups every three months for 2D echocardiography and check-up. A long-term study and follow-ups are required to determine the importance of this persistent winking coronary sign, as currently there is a lack of data to determine the significance of the sign in the patient’s long-term prognosis.
